# Study of laser fluorescence spectroscopy in livers of rats with hypothermic ischemia

**DOI:** 10.1590/acb386023

**Published:** 2023-12-01

**Authors:** Allison Takeo Tsuge, Jaqueline de Jesus Pereira, José Dirceu Vollet-Filho, Márcia Saldanha Kubrusly, Flávio Henrique Ferreira Galvão, Orlando Nascimento Ribeiro, Camila Rodrigues Moreno, Renata Nishiyama Ikegami, Eleazar Chaib, Orlando de Castro e Silva

**Affiliations:** 1 Universidade de São Paulo – Faculdade de Medicina de Ribeirão Preto – Departamento de Cirurgia e Anatomia –São Paulo(São Paulo), Brazil.; 2 Universidade de São Paulo – Hospital das Clínicas – Instituto do Coração – São Paulo (SP), Brazil.; 3 Universidade de São Paulo – Instituto de Física de São Carlos – São Carlos (SP), Brazil.; 4 Universidade de São Paulo – Hospital das Clínicas – Departamento de Gastroenterologia – São Paulo (SP), Brazil.; 5 Universidade de São Paulo – Hospital das Clínicas – Laboratório de Transplante e Cirurgia de Fígado – São Paulo (SP), Brazil.

**Keywords:** Liver, Spectrum Analysis, Fluorescence, Ischemia, Lasers, Hepatectomy

## Abstract

**Purpose:**

After partial hepatectomy (PH), the remaining liver (RL) undergoes regenerative response proportional to the host. Limited literature exists on hepatic viability after tissue injury during hypothermic preservation. Spectroscopy measures cellular fluorescence and is explored for tissue characterization and parameter investigation. This study aimed to assess fluorescence analysis (spectroscopy) in evaluating liver viability and its relationship with hepatic tissue regeneration 24 hours after PH. Additionally, we analyzed liver regeneration in RL after 70% partial hepatectomy under hypothermic conditions with laser irradiation.

**Methods:**

Fifty-six Wistar rats were divided into four groups: total non-perfused liver (control), total perfused liver, partial hepatectomy “in situ”, and partial hepatectomy “ex situ”. Tissue analysis was performed at 0 and 24 hours using spectroscopy with laser devices emitting at 532 (green) and 405 nm (violet).

**Results:**

Spectroscopy identified tissue viability based on consistent results with Ki67 staining. The fluorescence spectra and Ki67 analysis displayed similar patterns, linking proliferative activity and absorption intensity.

**Conclusions:**

Fluorescence spectroscopy proves to be promising for real-time analysis of cellular activity and viability. Metabolic activity was observed in groups of live animals and hypothermically preserved samples, indicating cellular function even under blood deprivation and hypothermic conditions.

## Introduction

The liver performs an important role because of its crucial metabolic functions such as protein synthesis and biotransformation of xenobiotics, besides the extraordinary regenerative capacity^1^. Hepatic regeneration (HR) represents a natural protection mechanism against loss of functional tissue and occurs mainly because of the restoration of liver mass through hyperplastic or hypertrophic compensation in the remaining lobes, with a consequent increase in size, and a growth controlled by functional or anatomical factors^2^^,^ 3.

The HR is a process highly harmonized by physiological factors including growth factors, such as hepatocyte growth factor, epidermal growth and fibroblast growth factor and proliferation of immune system cells^4^^,^ 5.

The knowledge acquired to understand how the HR process works originates from rodent models, in which two-thirds partial hepatectomy is performed^3^. In adult livers, after 70% hepatectomy, there is a process of hypertrophy of hepatocytes with replication in the remaining lobes and consequent restoration of metabolic activity. However, there is permanent loss of morphology and architecture of the injured lobe^6^.

After partial hepatectomy, the remaining liver (RL) needs to adapt hemodynamically, as it starts to receive all arterial and portal flow, which before partial hepatectomy irrigated the entire liver^4^^–^ 6. This causes metabolic overload in the RL and appears to trigger the regenerative process^7^^–^ 9.

The evaluation of liver viability through normothermic or hypothermic perfusion techniques can be introduced into clinical practice as beneficial conditions^10^. Croce et al.^11^ observed that preservation conditions and cold hypoxia induce an increase in signal amplitude, attributed to changes in nicotinamide adenine dinucleotide phosphate (NADPH), flavins and coenzymes involved in energy metabolism. Currently, all effective methods for preserving organs for transplantation are based on temperature reduction as the main protective element^12^^–^ 15.

There are different factors involved in the liver regeneration process and several methods capable of evaluating this phenomenon, such as the determination of liver mass (liver weight), mitosis count, DNA synthesis and immunohistochemical methods for detection of endogenous molecules as proliferating cell nuclear antigen (PCNA), Ki67 antigen, among others. Ki67 is a nuclear antigen expressed in the G1, S, G2 and M phases of the cell cycle. Its evaluation is used to quantify proliferative cell activity, and it is currently considered the gold standard for this purpose^14^^,^ 15.

The laser therapy is a practice that have been explored as a potential regenerative treatment^16^^–^ 18. Researchers have reported the regenerative capacity of laser irradiation of different wavelengths due to an important physiological effect, such as the induction of cell proliferation. The application of low-power laser can provide favorable HR after partial hepatectomy from the emergence of new hepatocytes, mesenchymal stem cells and angiogenesis in rat livers^19^^–^ 22.

Low-level laser therapy consists of the use of photons with a wavelength near infrared region and includes monochromatic diode lasers of relatively low power (below 500 mW)–this practice has been used in athermanous treatments because of its ability to not heat the biological tissues^19^^,^ 22.

All cells exhibit intrinsic natural autofluorescence, because of the presence of different cellular components such as flavins, nicotinamide-adenine-dinucleotide (NAD), aromatic amino acids, lipofuscins, advanced glycation end products, and collagen^20^. Autofluorescence is detectable from various biomolecules which act as endogenous fluorophores and can provide certain relevant information about pathophysiological conditions^21^. Also, cellular autofluorescence spectra cover most of the spectral range, as different endogenous fluorophores emit different wavelengths, such as flavins, NAD, and lipofuscin emit green, blue, and orange light, respectively, when excited at appropriate wavelengths^22^.

The intrinsic properties of liver tissue are strongly correlated with the fluorescence spectrum, in an attempt to assess metabolic activity^15^^,^ 23. Among these properties, we can highlight biomolecules such as NADPH, flavins, proteins, amino acids with aromatic side chains and porphyrins^24^^,^ 25. Thus, spectroscopy has the potential to provide important information about tissues through its ability to measure the fluorescence emitted by target cellular components, once they are adequately stimulated.

Therefore, our main objective was to evaluate the applicability of fluorescence spectroscopy analysis on liver viability in experimental models and its relationship with liver tissue regeneration 24 hours after partial hepatectomy.

As a secondary objective, we analyzed hepatic regeneration of the remaining liver after 70% partial hepatectomy in experimental models, in hypothermic conditions, to verify if irradiation with laser light improves the regeneration process.

## Methods

Male Wistar rats from the Central Animal Laboratory of the Medicine Faculty (Universidade de São Paulo, São Paulo, SP, Brazil), approximately weighing 300 g, were used in the present study. Animals were placed in transparent polypropylene cages, appropriate to the species, and maintained with specific food for the species and water *ad libitum*, with light and dark intervals of 12 hours.

Sample size was calculated according to the Kelley and Maxwell’s method^24^. Rats included in the study were randomly divided into four groups. In all groups, samples were placed in plastic bottles with 30 mL of static preservation solution (SPS)-1 (Belzer UW) at 4°C for 24 hours (except G4, whose samples were kept in the live rats). Half of the animals in all groups (n = 7) received irradiation of laser light (+L).

- G1: total non-perfused liver control (TNPL, n = 14): total hepatectomy was performed without perfusion with SPS-1;
- G2: total perfused liver (TPL, n = 14): hepatic perfusion was performed with SPS-1, followed by total hepatectomy;
- G3: partial perfused hepatectomy “ex situ” (PPH, n = 14): hepatic perfusion was performed with SPS-1. Then, partial hepatectomy was performed, and the left lateral lobe (which corresponds to 30% of the liver) was placed in plastic bottles for 24 hours following the protocol of this study;
- G4: partial non-perfused hepatectomy “in situ” (PNPH, n = 14): partial hepatectomy was performed, and only the left lateral lobe remained in the animals (30% of the liver). Rats were kept alive for 24 hours.

Animals in all groups were maintained and euthanized at the Central Animal Laboratory of the Faculty of Medicine, Universidade de São Paulo, where the samples were harvested for further study.

All procedures involving experimentation on animals were performed in accordance with Guide for the Care and Use of Laboratory Animals (1996, published by National Academy Press). Animal research protocol was approved by the local ethical review committee of the Universidade de São Paulo (Research protocol No 107/14; CEUA-FMUSP).

### Surgical procedures

To perform the surgical techniques, all animals received anesthesia by administering intraperitoneally (ketamine, 25–50 mg/kg associated with xylazine 2–5 mg/kg) at the dose of 15 units/100 g per weight.

**Figure 1:**
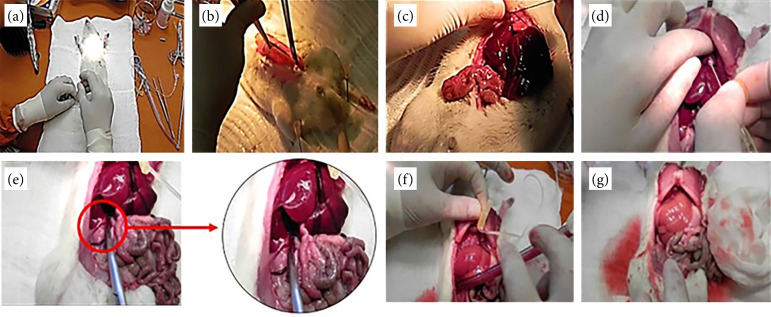
Hepatectomy in experimental surgery. **(a)** Pelage trimming; **(b)** opening of the abdomen; **(c)** 70% hepatectomy; **(d)** left ventricle puncture; **(e)** section of the infrahepatic vena cava; **(f)** perfusion with SPS-1 solution; **(g)** liver totally perfused with SPS-1.

Source: Elaborated by the authors.

Ph at 70% was performed according to Higgins and Anderson^25^. The procedure started with an abdominal incision in the midline. At this moment, liver tissue fragments were collected for histological analysis and preserved in 10% buffered formalin (Fig. 1).

Hepatic perfusion with Belzer UW solution was performed in TPL and PPH groups by hydrostatic pressure with thoracic access through diaphragmatic route, followed by puncture of the left ventricle with a needle catheter (Abocath, Becton Dickinson Indústria Cirúrgica, Brazil), 22 gauge, connected to a liquid column with 250 mL of perfusion solution, Belzer UW, 1.30-m tall, which corresponds to a pressure of approximately 90 mmHg. After the puncture, the infrahepatic vena cava was opened, and, finally, the perfusion itself was performed (Fig. 1).

Once the hepatic blood was exchanged for Belzer UW solution, the perfusion was interrupted, and the total or partial 70% hepatectomy was performed. During the operation, blood clots were thoroughly cleaned with gauze, and strict hemostasis was performed. An electric scalpel was used for cauterization when necessary. Specifically, for the PNPH group, the median incision was sutured with 4-0 nylon.

The animals that were kept alive for 24 hours received tramadol as a postoperative analgesic, and a 10% povidone-iodine antiseptic solution was applied over the surgical wound.

Liver fragments collected from total and partial hepatectomy were placed in plastic bottles with 30 mL of Belzer UW solution at 4°C for 24 hours, for later analysis.

The animals were euthanized by administering intramuscular anesthetic (ketamine, 25–50 mg/kg associated with xylazine 2–5 mg/kg) in lethal dose. Animals were immediately subjected to necropsy.

### Fluorescence measurement

Fluorescence measurement was processed according to the protocol of Castro e Silva et al.^26^ A spectroscopy system assembled with doubled -frequency Nd:YAG laser device emitting wavelength at 532nm and a diode laser emitting at 405nm as excitation sources was used. The system has a Y-type investigational probe (Ocean Optics, United States of America) with two 600-µM optical fibers, one providing the laser excitation for fluorescence and the other one collecting the re-emitted light from the target tissue. A 550-nm high-pass filter (OGG550, Schott, United States of America) was used to remove backscattered light before entering the spectrometer for 532 nm excitation, and one at 450 nm for 405 nm excitation.

Analyses were performed at two different wavelengths, the first one at 405 nm (violet) and the second one at 532 nm (green). For each wavelength, four measurements of the left lateral lobe were performed, totaling eight analyses. We carried out an initial reading, called internal control, that is, the analysis of the livers before tissue injury. Next, we performed an analysis of the time 0 hour (T0), and, in the groups in which there was perfusion with SPS-1, this reading occurred right after the perfusion, and before the application of laser therapy.

After 24 hours (T24) of the experiment, new spectrometry analyses were performed, in the same format. In each group of 14 animals, seven had the liver irradiated with laser therapy at T0.

### Laser therapy

Half of the animals of each group (n = 7) were irradiated with the Laser CW (model BDP 660), red light wavelength 660 nm, class IIIb, continuous emission of visible light, low power, with semiconductor active medium and fused optical fiber transducer with 80 mm in length and diameter of 7 mm, produced by M.M. Optics (São Carlos, São Paulo, Brazil), for irradiation of livers, in application of laser light in five consecutive sessions of 60 seconds (total 300 seconds), with each application in a different point of the liver. Each point was arbitrarily chosen, but, once chosen, this pattern was followed in all irradiated livers, putting the distal end of the transducer in contact with the liver surface (calibration at a power of 40 mW, with an energy density dose of 300 J/cm^2^), performing high penetrability in the biological tissue^27^^,^ 28.

### Analysis of fluorescence spectroscopy data

For acquisition and storage of wavelengths, a laptop coupled to the spectrophotometer was used, and the software used was OOIBase32.exe (Ocean Optics). The resulting data were exported to an analysis software (OriginPro 8) for compilation, normalization of results and obtaining the graphics.

### Immunohistochemistry

Five-micron serial sections of formalin-fixed, paraffin-embedded tissue were cut for immunohistochemical detection of antigens from Ki67. Antigen retrieval was performed in a pressure cooker by heating the sections with citrate buffer, followed by endogenous peroxidases block with 6% hydrogen peroxide. The sections were then incubated with a serum-free protein block (CAS Block, Invitrogen-008120) to inhibit non-specific binding, and then with primary antibody against Ki67 (1:100; rabbit clone SP6 – ab 16667; Abcam, Cambridge, United Kingdom) for 1 hour. The MACH 4 polymer (Universal HRP-Polymer Detection System, BIOCARE Medical-BRI4012) was used for detection of the primary antibody. The staining procedure was completed using diaminobenzidine (DAB; Sigma Chemical Corporation, St. Louis, MO, United States of America) as a chromogen. Finally, the sections were counterstained with Harris’ hematoxylin.

The number of positive cells/40× (an average of 20 fields) was quantified to define the average number of immune-stained cells. Results were expressed as cells/mm^2^, and this number was divided by the value of 0.159, which is the diameter of the microscopic field at 40x magnification.

### Statistical analysis

The quantitative variables obtained were expressed as median and interquartile range. Statistical analysis was performed using GraphPad Prism 6. The Mann-Whitney’s test was used for unpaired variables with asymmetrical distributions. We considered p < 0.05 statistically significant.

## Results

### Cellular proliferation

**Figure 2:**
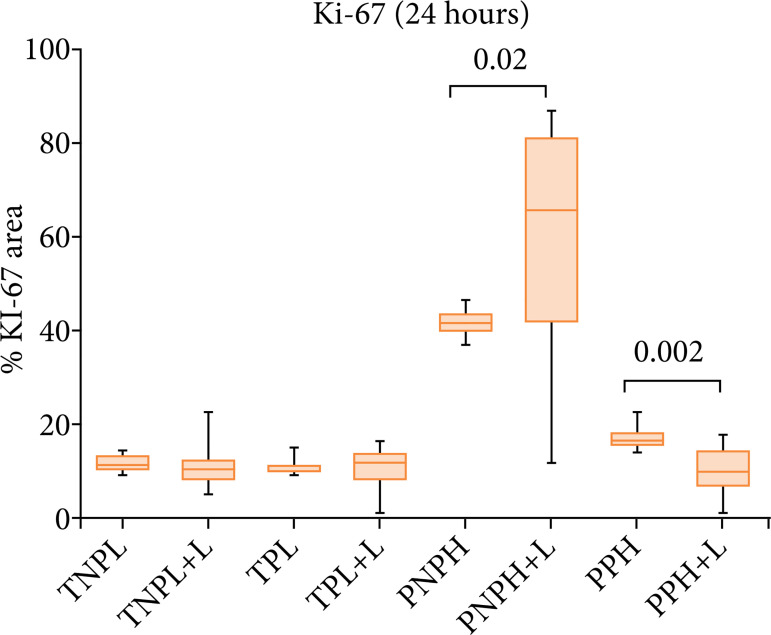
Quantification and comparison of Ki67 between groups, demonstrating higher expression of the protein in the PNPH-/+L and PPH groups.

TNPL: total non-perfused liver control; TPL: total perfused liver; PNPH: partial non-perfused hepatectomy “in situ”; PPH: partial perfused hepatectomy “ex situ”. Source: Elaborated by the authors.

There was predominance of Ki67 (p = 0.02) in the PNPH +L, compared with the PNPH group. In the PPH group, there was an increased level of Ki67 when compared with the PPH + L. These differences were absent in the TNPL, TNPL +L, TPL and TPL +L groups (Fig. 2).

### Spectrometric analysis

We considered the animals from all groups at T0 as a control group. This group played the role of internal control for the fluorescence collection obtained by the spectrophotometer.

**Figure 3:**
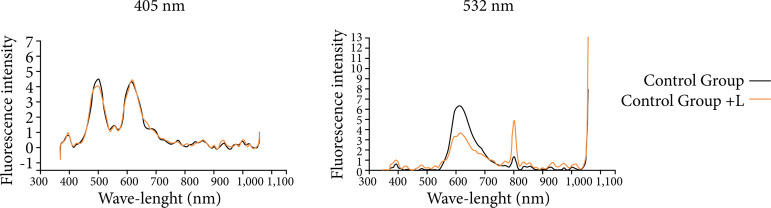
Fluorescence spectrum of the left lateral lobe of the animals in the control group, showing overlapping of the curves of the studied wavelengths, 405 and 532 nm.

Source: Elaborated by the authors.

The analysis of the control group showed differences regarding the intensity of fluorescence emission. Two intense peaks were observed between 450–650 nm, when excited at the length of 405 nm (Fig. 3).

**Figure 4:**
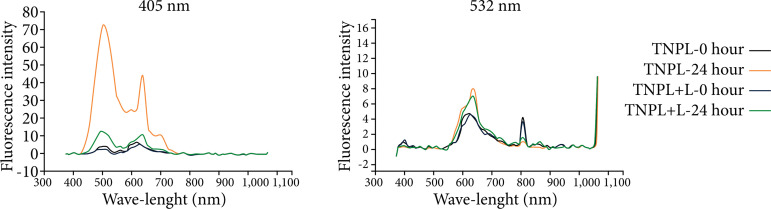
Fluorescence spectrum of the left lateral lobe of the animals in the TNPL group, showing the curves of time 0 and time 24 hours, with wavelengths of 405 and 532 nm.

**Figure 5:**
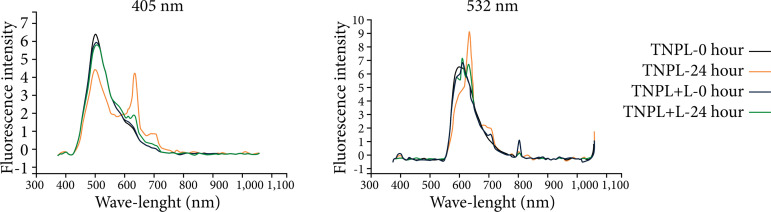
Fluorescence spectrum of the left lateral lobe of the animals in the TPL group, showing the curves of time 0 and time 24 hours, with wavelengths of 405 and 532 nm.

**Figure 6:**
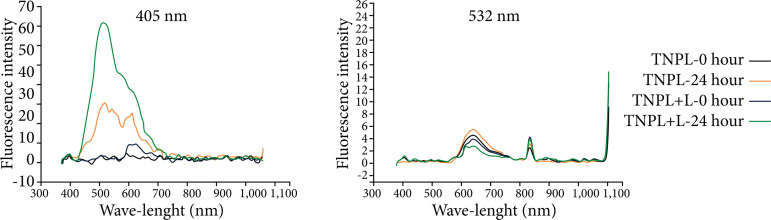
Fluorescence spectrum of the left lateral lobe of the animals in the PNPH group, showing the curves of time 0 and time 24 hours, with wavelengths of 405 and 532 nm.

**Figure 7:**
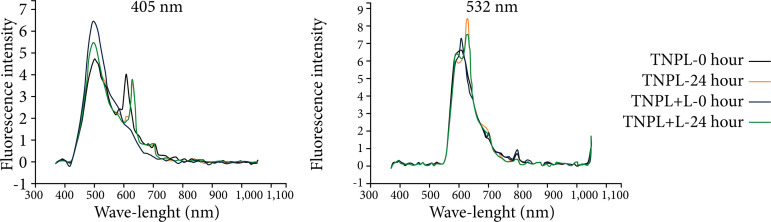
Fluorescence spectrum of the left lateral lobe of the animals in the PPH group, showing the curves of time 0 and time 24 hours, with wavelengths of 405 and 532 nm.

**Figure 8:**
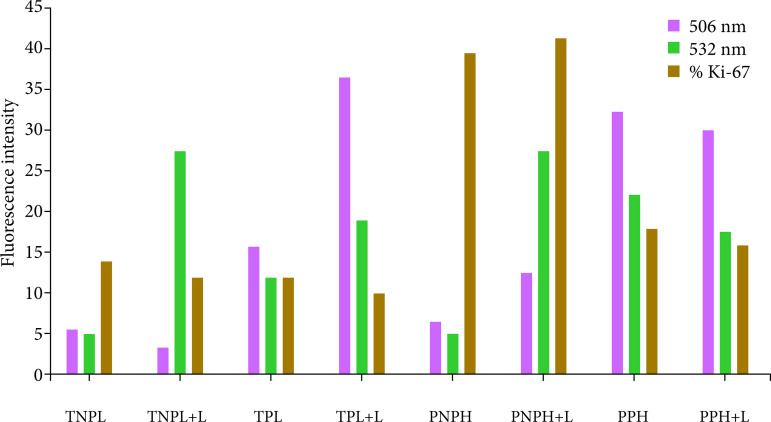
Comparison of fluorescence spectrophotometer analysis at wavelengths of 405 and 532 nm with Ki67 immunohistochemical analysis.

TNPL: total non-perfused liver control; TPL: total perfused liver; PNPH: partial non-perfused hepatectomy “in situ”;PPH: partial perfused hepatectomy “ex situ”. Source: Elaborated by the authors.

When we compare the analyses performed by the spectrophotometer in the study groups, with or without the use of laser light, there is an overlap of the fluorescence intensity curves, in both wavelengths used in the methodology (405–532 nm). All groups showed a spectral range between 400–600 nm, with higher peak light absorption intensity at 24 hours (Figs. 4–8).

TNPL: total non-perfused liver control. Source: Elaborated by the authors.

TPL: total perfused liver control. Source: Elaborated by the authors.

PNPH: partial non-perfused hepatectomy “in situ”. Source: elaborated by the authors.

PPH: partial perfused hepatectomy “ex situ”. Source: Elaborated by the authors

## Discussion

Autofluorescence spectroscopy is a technique that has been increasingly used as a technical potential for tissue characterization, with diagnostic and therapeutic purposes^29^.

The absorption of laser light by tissues can result in four basic effects, such as photochemical, photothermal, photomechanical and photoelectric, depending on the wavelength used and its penetration capacity. Therapeutic lasers can activate or inhibit physiological, biochemical, and metabolic processes^30^.

Thus, there are several biomolecules involved both in metabolic processes and in the histological organization of cells and tissues, which are characterized by their fluorescence properties, and can be explored to obtain information on the morphofunctional conditions of the studied organ^31^.

In the analysis of the control group, we observed that there were two intense peaks in the spectral ranges of 500 and 620 nm, when the tissue was excited at the length of 405 nm.

Fluorescence spectra in this range infer changes related to biological events in the tissue, since molecules such as nicotinamide adenine dinucleotide (NADH) and flavin adenine dinucleotide (FAD) and porphyrins emit fluorescence in lengths of 465, 535 and 630–690 nm, respectively^32^.

NADH and FAD molecules can provide information about the redox status of cells and tissues. Porphyrins, on the other hand, correspond to compounds present in hemoglobin, such as heterocyclic rings^33^. Porphyrin derivatives, from the biosynthesis of the iron heme, present in red blood cells, have excitation wavelength at 405 nm, emission at 630–690 nm, giving them the natural characteristic of their pigment in purple^25^.

When we compared the information obtained by the spectrophotometer, there was an overlap of the fluorescence intensity curves in both wavelengths used in the methodology (405 and 532 nm). All groups presented spectral range between 400 to 700 nm, with peak intensity of light emission higher in the period of 24 hours, both in animals that were irradiated, as well as in those that did not receive stimulation with laser light.

This corroborates with data present in the literature, since DNA molecules and proteins are the main ultraviolet (UV)-absorbing chromophores, from 200 to 400 nm, in biological tissue, inferring active cellular activity^34^^,^ 35.

The biochemical constitution of tissue and blood is largely responsible for the spectral characteristics of fluorescence, since blood is a great absorber of green light (532 nm). That is, while amplitude has great relationship with absorption and penetrability, the shape of the spectrum, such as peaks and widths, has to do with the chemical constitution of the organ in question^36^. The spectral absorption range between 400 and 600 nm is related to oxyhemoglobin, hemoglobin and melanin^37^.

These data are more expressed in the TNPL and PNPH groups, in which both underwent surgical stress, but only in the latter the animals were kept alive 24 hours after the procedure. In the spectrophotometric analysis of the PNPH group, it seems, there was a substantial increase for the PNPH +L subgroup. These were probably because the liver tissue suffered injury (70%) hepatectomy associated with the fact that the hepatotrophic factors necessary to increase cell activity were maintained. We observed that, even without providing energy in the form of laser light, there was an important increase in cellular activity, which was optimized to the extent that laser light was provided to the remaining liver. Ki67 analysis seems to corroborate the spectrophotometer data, presenting a significant difference between these subgroups (p = 0.02).

Thus, we can infer that by stimulating tissue after partial hepatectomy with laser light there is a bio-stimulating action to promote tissue regeneration, since Ki67 is a nuclear protein associated with the cell cycle, synthesized by all proliferating cells. Some studies report the action of low-level laser therapy as a potential factor involved with numerous genes connected to mitochondrial signaling, cell binding, cell proliferation and differentiation, in addition to collagen synthesis^38^^,^ 39.

Barbosa et al.^40^ suggest that in 24 hours after exposure to low-density laser there is an important increase in ATP production. In addition, some authors demonstrate that the application of laser therapy can increase mitotic index and mitochondrial function in RL after hepatectomy of different levels^21^^,^ 41.

In the PPH group, fluorescence spectrophotometry analysis showed a difference of the spectra between groups. Ki67 analysis seems to correspond to the findings of spectroscopy with statistically significant difference (p = 0.002). Apparently, there was a drop-in cell activity in the PPH +L subgroup, despite being a group in which liver injury is present, as the liver samples were free of hepatotrophic factors, since they were submerged with SPS-1. Because this group receives a surplus of energy in the form of laser light, apparently the hepatic tissue only increases if its cellular activity is stimulated by metabolic action, impinged by blood and portal pressure of the host.

The TPL group, on the other hand, is not influenced by the blood or the host. Therefore, it does not receive the hepatotrophic stimuli that commonly come from them. The blood was completely replaced by the SPS-1, and there was no tissue damage. Spectrophotometric data apparently show that both groups, with and without stimulation with laser light, are very similar, showing that only the supply of extra energy (laser light) is not enough for there to be appreciable liver regeneration.

In general, both the data from the fluorescence spectra and the Ki67 analysis showed the same pattern of results, with the increase in proliferative activity being proportional to the intensity of light absorbance, suggesting the quality of the spectrophotometric identification, so that we can say that we maintained a consistent standard when compared to Ki67, the gold standard of analysis.

## Conclusion

The data presented up to now are promising, since fluorescence spectrometry apparently can be used for the analysis of characteristics of cell activity and viability in real time. As a second aspect, we can also infer the presence of metabolic activity, both in the groups of animals kept alive and in the hypothermal preserved samples, deducing that even with blood deprivation and in conditions of hypothermia after 24 hours there is some cellular activity. When we maintain hepatotrophic factors associated with the application of tissue injury, the supply of energy in the form of laser light to the hepatic tissue seems to increase the metabolic activity and, therefore, the regenerative process.

## Data Availability

All data sets were generated or analyzed in the current study.
